# Molecular identification of *Aquilaria* species with distribution records in China using DNA barcode technology

**DOI:** 10.1080/23802359.2021.1914210

**Published:** 2021-04-26

**Authors:** Yong Kang

**Affiliations:** Hainan Provincial Key Laboratory of Resources Conservation and Development of Southern Medicine and Key Laboratory of State Administration of Traditional Chinese Medicine for Agarwood Sustainable Utilization, Hainan Branch of the Institute of Medicinal Plant Development, Chinese Academy of Medical Sciences and Peking Union Medical College, Haikou, China

**Keywords:** *A. sinensis*, *A. yunnanensis*, DNA barcoding, phylogenetic tree

## Abstract

*Aquilaria* species is one of the main plant resources that produce agarwood, which containing black resin with important economic and medicinal values. There are about 15 species known to the genus around the world, but only two can be found in China, i.e. *A. sinensis* and *A. yunnanensis*. In this study, *A. sinensis* and *A. yunnanensis* that endemic respectively to Hainan and Yunnan were sampled, on the basis of the investigation and observation of their main morphological features in plantation. Five primers, i.e. ITS2, *matK*, *trnL-trnF1, trnL-trnF2*, and *trnH-psbA*, were eventually selected for DNA barcoding. The results showed that the seed surface of *A. sinensis* is smooth or sparsely pubescent, and the seed appendages were long. While the seed surface of *A. yunnanensis* is densely covered with yellow hairs and the seed appendages are short. The *trnL-trnF1* sequence fragment has significant intraspecific and interspecific genetic distances. However, the species identification success rate of ITS2+*matK* combination was finally screened to be the highest, which was verified by the BBA method of TaxonDNA. The phylogenetic trees cluster analysis revealed that the classification of *A. sinensis* and *A. yunnanensis* is significant, and there is geographic isolation between the two species. Therefore, on the premise of accurate identification of plant morphological characters, ITS2+*matK* combination can be used to accurately identify the *Aquilaria* species in China.

## Introduction

*Aquilaria* species of Thymelaeaceae, the tropical and subtropical evergreen trees, that are mainly distributed in tropical or subtropical regions of the Southeast Asia, are the most important plant resources for the production of rare agarwood (FOC Eco 1999). Agarwood is a precious traditional medicinal ingredient and natural perfume in China, and it has been used widely for cultural, religious, and medicinal purposes around the world. In recent years, wild resources of *Aquilaria* species are found depleted due to the serious human logging and the destruction of their natural environments. *Aquilaria crassna* is listed as a critically endangered species by International Union for Conservation of Nature (IUCN), and *A. malaccensis*, as well as *A. sinensis*, are also regarded as vulnerable species (Hashim et al. 2016). In addition, all *Aquilaria* species have been included in the Convention on International Trade in Endangered Species (CITES) ((CITES) 2004).

Because of the high level of similarity in morphological features of *Aquilaria* species, previous identification methods are only dependent on the classifications of the different morphological features of flower, seed, and fruit. In addition, the flowering and fruiting period of *Aquilaria* species is unstable, and its wild resources are on the verge of extinction. Therefore, it is extremely difficult to identify the *Aquilaria* species by only collecting their fruits and flowers through field sampling, not to mention the high error generated (Lee and Mohamed 2016). In conclusion, this is also one of the factors that have not been clear about the taxonomic study of *Aquilaria* species. Several studies have shown that more than 20 *Aquilaria* species are distributed in the tropical regions of Southeast Asia (Lee et al. 2016; Gao et al. 2017). The Flora of Malaysia is one of the earliest Flora to describe the morphological characteristics of *Aquilaria* species (Hou 1960). And *Aquilaria* species are divided into 12 taxons in this flora, i.e. *A. malaccensis*, *A. microcarpa, A. brachyantha, A. urdanetensis, A. citrinaecarpa, A. apiculata, A. filaria, A. parvifolia, A. hirta, A. rostrata, A. beccariana,* and *A. cumingiana.* Over the past few years, *Aquilaria* species in Asian mainland were indicated that can be divided into 13 taxons (Santisuk 2007), i.e. *A. baillonii, A. banaensis, A. beccariana, A. crassna, A. hirta, A. khasiana, A. malaccensis, A. microcarpa, A. rostrata, A. rugosa, A. sinensis, A. subintegra,* and *A. yunnanensis*. However, about 15 *Aquilaria* species that discovered across the world were recorded in Flora of China (FOC Eco 1999), including *A. sinensis* and *A. yunnanensis*.

DNA barcoding has been proved the quick and accurate approach to identify different species based on selection of standard DNA segments (Hebert et al. 2003). This technology are also used widely for the identification of *Aquilaria* species. For example, *trnL-trnF* sequence was found that can provide new molecular framework for the identification of *Aquilaria* species (Eurlings and Gravendeel 2005). Similarly, phylogenetic trees constructed by *trnL-trnF*+ITS2 combination was demonstrated that is useful for identifying the *Aquilaria* species (Lee et al. 2016). Meanwhile, ITS sequence was applied in first time to analyze *A. malaccensis* from different sources (Lee et al. 2018). In addition, phylogenetic tree constructed by *trnL-trnF*+ITS1 was found that could aggregate the DNA sequence of *A. sinensis* in GenBank (Jiao et al. 2014). When comparing *A. sinensis*, *A. yunnanensis* and *A. crassna*, Li et al. found that the combinations of ITS+*matK* and ITS+*trnL-trnF* are suitable for molecular identification of these three species (Li et al. 2018). In the previous research, our group also found that *matK* fragments play an important role in *Aquilaria* species from multiple sources (Kang et al. 2019).

Although DNA barcoding is crucial in the identifications of various *Aquilaria* species, the selection of barcode fragments or combinations may be different for different materials. Moreover, DNA barcode technology combined with traditional classification features can obtain the best identification results on the basis of accurate collection of samples. Hence, 18 samples of *A. sinensis* and seven of *A. yunnanensis* were collected respectively from six plantations in Hainan and two plantations in Yunnan. Both *Aquilaria* species were studied on site during their flowering and fruit-bearing periods, and their morphological features were summarized. Five primers were selected for DNA barcodes study. On the premise of determining the morphological characteristics, it is planned to screen out the barcode fragments or combinations suitable for the identification and the construction of phylogenetic trees of both *Aquilaria species* by analyzing the sequence characteristics, genetic distances and species identification rates of different primers.

## Materials and methods

### Materials

A total of 25 samples of *A. sinensis* and *A. yunnanensis* that collected from 9 plantations in Hainan and Yunnan of China were used as the experimental materials. Fresh leaves were dried and preserved with silica gel, then extracted the total plant DNA. The voucher specimens were also deposited in the Herbarium of Traditional Chinese Medicine, Hainan Branch of the Institute of Medicinal Plant Development, Chinese Academy of Medical Sciences. Localities of all sampled accessions are shown in Table 1.

**Table 1.** Sample collection information and GenBank accession numbers of the *Aquilaria* species generated through this study.

Species	Collection number	Location	Region of origin (number of samples)	GenBank accession numbers				
				ITS2	*matK*	*trnL-trnF1*	*trnL-trnF2*	*trnH-psbA*
*A. sinensis*	HH0001	Tropical Medicinal Plant Garden, Haikou, IMPLAD	Hainan, China (3)	MW118060	MW118085	MW124309	MW124359	MW124334
	HH0002			MW118061	MW118086	MW124310	MW124360	MW124335
	HH0003			MW118062	MW118087	MW124311	MW124361	MW124336
*A. sinensis*	HX0001	Tropical Medicinal Plant Garden, Xinglong, IMPLAD	Hainan, China (3)	MW118063	MW118088	MW124312	MW124362	MW124337
	HX0002			MW118064	MW118089	MW124313	MW124363	MW124338
	HX0003			MW118065	MW118090	MW124314	MW124364	MW124339
*A. sinensis*	HC0001	Plantation, Fushan, Chengmai	Hainan, China (3)	MW118066	MW118091	MW124315	MW124365	MW124340
	HC0002			MW118067	MW118092	MW124316	MW124366	MW124341
	HC0003			MW118068	MW118093	MW124317	MW124367	MW124342
*A. sinensis*	HD0001	Plantation, Longhu, Dingan	Hainan, China (3)	MW118069	MW118094	MW124318	MW124368	MW124343
	HD0002			MW118070	MW118095	MW124319	MW124369	MW124344
	HD0003			MW118071	MW118096	MW124320	MW124370	MW124345
*A. sinensis*	HW0001	Plantation, Maoyang, Wuzhishan	Hainan, China (3)	MW118072	MW118097	MW124321	MW124371	MW124346
	HW0002			MW118073	MW118098	MW124322	MW124372	MW124347
	HW0003			MW118074	MW118099	MW124323	MW124373	MW124348
*A. sinensis*	HY0001	Plantation, Yanfeng, Haikou	Hainan, China (3)	MW118075	MW118100	MW124324	MW124374	MW124349
	HY0002			MW118076	MW118101	MW124325	MW124375	MW124350
	HY0003			MW118077	MW118102	MW124326	MW124376	MW124351
*A. yunnanensis*	YML0001	Baihuashan, Mengla, Xishuangbanna	Yunnan, China (3)	MW118078	MW118103	MW124327	MW124377	MW124352
	YML0002			MW118079	MW118104	MW124328	MW124378	MW124353
	YML0003			MW118080	MW118105	MW124329	MW124379	MW124354
*A. yunnanensis*	YMY0001	Nabanhe, Mengyang, Xishuangbanna	Yunnan, China (3)	MW118081	MW118106	MW124330	MW124380	MW124355
	YMY0002			MW118082	MW118107	MW124331	MW124381	MW124356
	YMY0003			MW118083	MW118108	MW124332	MW124382	MW124357
*A. yunnanensis*	YB0001	Xishuangbanna Tropical Botanical Garden, Chinese Academy of Sciences	Yunnan, China (1)	MW118084	MW118109	MW124333	MW124383	MW124358

### Methods

#### Observation of the main morphological features of Aquilaria species

The six plantations in Hainan and two plantations in Yunnan were investigated. Description of the main reproductive organ characteristics, such as fruits and seeds of *A. sinensis* and *A. yunnanensis* by collecting samples of the *Aquilaria* species. And it was made into wax leaf specimens for preservation.

#### DNA extraction, amplification and sequencing

The total DNA extraction kit for plants that acquired from Tiangen Biotech (Beijing) Co., Ltd was used to extract the DNA, and five primers (i.e. ITS2, *matK*, *trnL-trnF1*, *trnL-trnF2* and *trnH-psbA*) were used for PCR amplification (Table 2). Optimization and adjustment were made based on the previously reported PCR reaction system (Group et al. 2009). The sequencing of all amplification products was completed by Guangzhou IGE Biotechnology Ltd. Bioedit (Hall 1999), Sequencematrix (Vaidya et al. 2011), Mega X (Sudhir et al. 2018), MrBayes 3.2.6 (Huelsenbeck and Ronquist 2001) and PAUP 4 b (http://paup.phylosolutions.com) were used to edit and compare the sequences, match the barcodes, calculate the genetic distances and build the phylogenetic trees, while TaxonDNA (Meier et al. 2006) was used to calculate species identification rates, and R 4.0.0 (https://www.r-project.org) and Figtree 1.4.3 (http://tree.bio.ed.ac.uk/software/figtree/) were used to beautify the phylogenetic trees.

**Table 2.** Details on the PCR primers used in this study.

DNA barcode	Primer	Primer sequence (5’-3’)	PCR reaction conditions
ITS2 (Chen et al. 2010)	ITS-S2F	ATGCGATACTTGGTGTGAAT	94 °C 5 min; 94 °C 30 s, 56 °C 30 s, 72 °C 45 s, 40 cycles;72 °C 10 min;4 °C save.
	ITS-S3R	GACGCTTCTCCAGACTACAAT	
*matK* (Kim, unpublished)	3F_KIM	CGTACAGTACTTTTGTGTTTACGAG	94 °C 1 min; 94 °C 30 s, 52 °C 20 s, 72 °C 50 s, 35 cycles;72 °C 5 min;4 °C save.
	1R_KIM	ACCCAGTCCATCTGGAAATCTTGGTTC	
*trnL-trnF1* (Lee et al. 2016)	e	GGTTCAAGTCCCTCTATCCC	94 °C 5 min; (94 °C 45 s, 50 °C 45 s, 72 °C 90 s, 30 cycles);72 °C 10 min;4 °C save.
	f	ATTTGAACTGGTGACACGAG	
*trnL-trnF2* (Lee et al. 2016)	F-forw-2	CAAATCAACATTTTTGAGTAAGGAA	94 °C 5 min; (94 °C 20 s, 52–55 °C 20 s, 72 °C 45 s, 35 cycles);72 °C 5 min;4 °C save.
	E-Aq-rev-1	CGAACGGGAATTGACAGAAT	
*trnH-psbA* (Sang et al. 1997; Tate and Simpson 2003)	trnHf_05	CGCGCATGGTGGATTCACAATCC	94 °C 5 min; (94 °C 1 min, 55 °C 1 min, 72 °C 90 s, 30 cycles);72 °C 7 min;4 °C save.
	psbA3-f	GTTATGCATGAACGTAATGCTC	

#### Data analysis

The rate of PCR amplification can be defined as the ratio in the percentage of the number of successful individuals to the total number of individuals while sequencing success rate is the percentage of the number of high-quality sequences obtained to the total number of sequences (Kress et al. 2009). MEGA X was used to compare multiple sequences, calculate sequence length, variable sites and conserved sites. Intraspecific and interspecific genetic distances were calculated by K2P model of MEGA X. The ‘Best match,’ ‘Best close match’ and ‘All species barcodes’ (BBA method) in TaxonDNA software were used to evaluate the success rates of species identification and to screen for the best DNA fragments or combinations. Moreover, the phylogenetic trees were generated using the neighbor-joining (NJ) and Unweighted Pair-group Method with Arithmetic Mean (UPGMA) methods in MEGA X, with individual node support calculated based on 1000 bootstrap re-samplings. In addition, Bayesian interference (BI) and Maximum Likelihood (ML) approaches were also chosen for the construction of phylogenetic trees in MrBayes 3.2.6 and PAUP 4 b, respectively.

## Results

### Main morphological features of A. sinensis and A. yunnanensis

**Figure 1.**
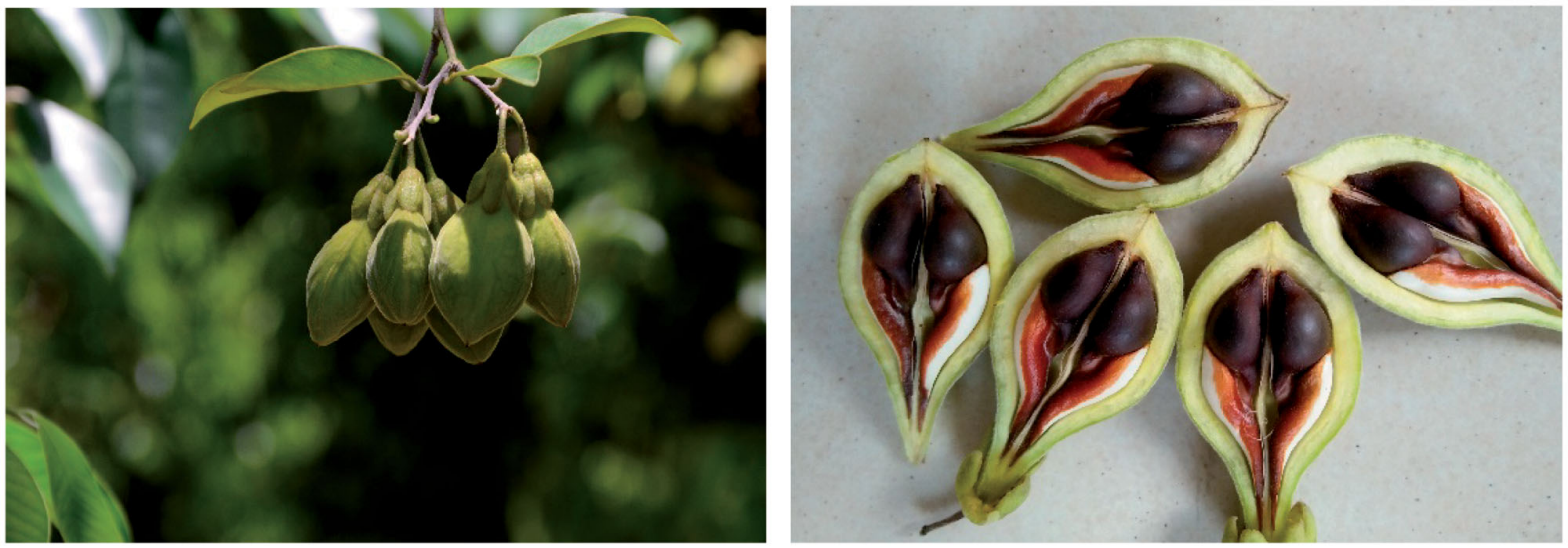
Fruits and seeds of *A. sinensis*.

**Figure 2.**
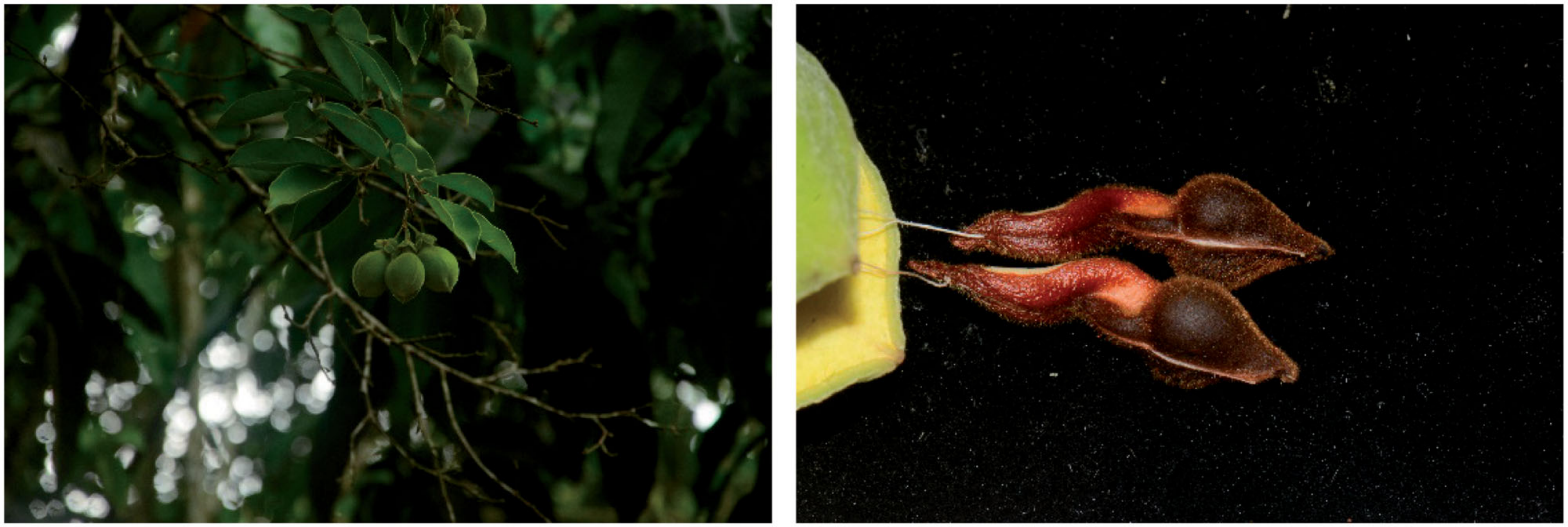
Fruits and seeds of *A. yunnanensis*.

Fruit of *A. sinensis* is oblong, with a long beak, moderate calyx without wrapping the fruit, and its seed surface is smooth or sparsely covered with white pubescence, with long appendages. However, fruit of *A. yunnanensis* is oval, with small and scattered calyx, short seed appendages, and the seed surface is densely covered with yellow pubescence. In conclusion, the main distinguishing characteristics between *A. sinensis* and *A. yunnanensis* are whether the seed surface is densely covered with yellow pubescence and the length of seed appendages (Figures 1 and 2).

### Distinguishing both Aquilaria species using DNA barcoding

#### Sequence characteristics of the DNA barcodes

PCR amplification and sequencing were implemented for ITS2, *matK*, *trnL-trnF1*, *trnL-trnF2* and *trnH-psbA* sequences of all samples to obtain the corresponding rates of PCR amplification, sequencing rates, and variable sites data (Table 3). It could be observed from the Table3 that the rates of PCR amplification and sequencing rates of above 5 sequences were 100.00%. Ordering their lengths in a descending order, *matK* (775) > ITS2 (456) = *trnL-trnF1* (456) > *trnH-psbA* (408)> *trnL-trnF2* (115).The number of variant sites from highest to lowest is *trnL-trnF1* (11) > ITS2(2) > *matK* (1) > *trnL-trnF2* (0) *= trnH-psbA* (0), while the number of conserved sites from highest to lowest is *matK* (769) > ITS2 (450) > *trnL-trnF1* (439） > *trnH-psbA* (407) > *trnL-trnF2* (115).The number of singleton sites in *trnL-trnF1* was 4, and the rest of the fragments were 0.

**Table 3.** Evaluation of the five DNA barcode loci and their combinations.

DNA barcode	PCR success (%)	Sequencing success (%)	Sequence length	No. of variable/Conserved sites	No. of parsimony informative sites	No. of singleton sites
ITS2	100	100	456	2/450	2	0
*matK*	100	100	775	1/769	1	0
*trnL-trnF1*	100	100	456	11/439	7	4
*trnL-trnF2*	100	100	120	0/115	0	0
*trnH-psbA*	100	100	408	0/407	0	0
ITS2+*matK*	–	–	1231	3/1219	3	0
ITS2+*trnL-trnF1*	–	–	912	13/889	9	4
ITS2+*trnL-trnF2*	–	–	576	2/565	2	0
ITS+*trnH-psbA*	–	–	864	2/857	2	0
*matK*+*trnL-trnF1*	–	–	1231	12/1208	8	4
*matK*+*trnL-trnF2*	–	–	895	1/884	1	0
*matK*+*trnH-psbA*	–	–	1183	1/1176	1	0
*trnL-trnF1*+*trnL-trnF2*	–	–	576	11/554	7	4
*trnL-trnF1*+*trnH-psbA*	–	–	864	11/846	7	4
*trnL-trnF2*+*trnH-psbA*	–	–	528	0/522	0	0
ITS2+*matK*+*trnL-trnF1*	–	–	1687	14/1658	10	4
ITS2+*matK*+*trnL-trnF2*	–	–	1351	3/1334	3	0
ITS2+*matK*+*trnH-psbA*	–	–	1639	3/1626	3	0
*matK*+*trnL-trnF1*+*trnL-trnF2*	–	–	1351	12/1323	8	4
*matK*+*trnL-trnF1*+*trnH-psbA*	–	–	1639	12/1615	8	4
*trnL-trnF1*+*trnL-trnF2*+*trnH-psbA*	–	–	984	11/961	7	4
ITS2+*trnL-trnF1*+*trnL-trnF2*	–	–	1032	13/1004	9	4
ITS2+*trnL-trnF1*+*trnH-psbA*	–	–	1320	13/1296	9	4
ITS2+*trnL-trnF2*+*trnH-psbA*	–	–	984	2/972	2	0
*matK*+*trnL-trnF2*+*trnH-psbA*	–	–	1303	1/1291	1	0
ITS2+*matK*+*trnL-trnF1*+*trnL-trnF2*	–	–	1807	14/1773	10	4
ITS2+*matK*+*trnL-trnF1*+*trnH-psbA*	–	–	2095	14/2065	10	4
*matK*+*trnL-trnF1*+*trnL-trnF2*+*trnH-psbA*	–	–	1759	12/1730	8	4
ITS2+*matK*+*trnL-trnF2*+*trnH-psbA*	–	–	1759	3/1741	3	0
ITS2+*trnL-trnF1*+*trnL-trnF2*+*trnH-psbA*	–	–	1440	13/1411	9	4
ITS2+*matK*+*trnL-trnF1*+*trnL-trnF2*+*trnH-psbA*	–	–	2215	14/2180	10	4

#### Genetic distance

The K2P distance model was selected by MEGA X software to calculate the interspecific and intraspecific genetic distances among five fragments and their combinations. The results are as shown in Supplementary Table 1. For *A. sinensis*, the range of intraspecific genetic distances among the five primers and their combinations was 0.000–0.016%, while the range of average intraspecific genetic distances was 0.000–0.007%. For *A. yunnanensis*, the range of intraspecific genetic distances among the five primers and their combinations was 0.000–0.016%, while the range of average intraspecific genetic distances was 0.000–0.006%. Moreover, the range of interspecific genetic distances between two species was 0.000–0.016%, while the range of average intraspecific genetic distances was 0.000–0.006%. To summarize, this study found that *trnL-trnF1* has the maximum average intraspecific and interspecific genetic distances, followed by ITS2.

#### Species identification

This paper used the ‘BBA’ method in TaxonDNA to verify and analyze the identification rates for the *Aquilaria* species, and the results are as shown in Supplementary Table 2. The best performing single fragment in ‘best match’ and ‘best close match’ was *matK,* with an accurate identification rate of 81.00%. Some of the best performing multi- fragment combinations in ‘best match,’ ‘best close match,’ and ‘all species’ include ITS2+*matK* (96.00%), *matK*+*trnL-trnF2* (81.00%), *matK*+*trnH-psbA* (81.00%), ITS2+*matK*+*trnL-trnF1* (84.00%), ITS2+*matK*+*trnL-trnF2* (96.00%), ITS2+*matK*+*trnH-psbA* (96.00%), *matK*+*trnL-trnF2*+*trnH-psbA* (81.00%), ITS2+*matK*+*trnL-trnF1*+*trnL-trnF2* (84.00%), ITS2+*matK*+*trnL-trnF1*+*trnH-psbA* (84.00%), ITS2+*matK*+*trnL-trnF2*+*trnH-psbA* (96.00%), and *trnL-trnF1*+*trnL-trnF2*+*trnH-psbA* (84.00%).

#### Phylogenetic tree

**Figure 3.**
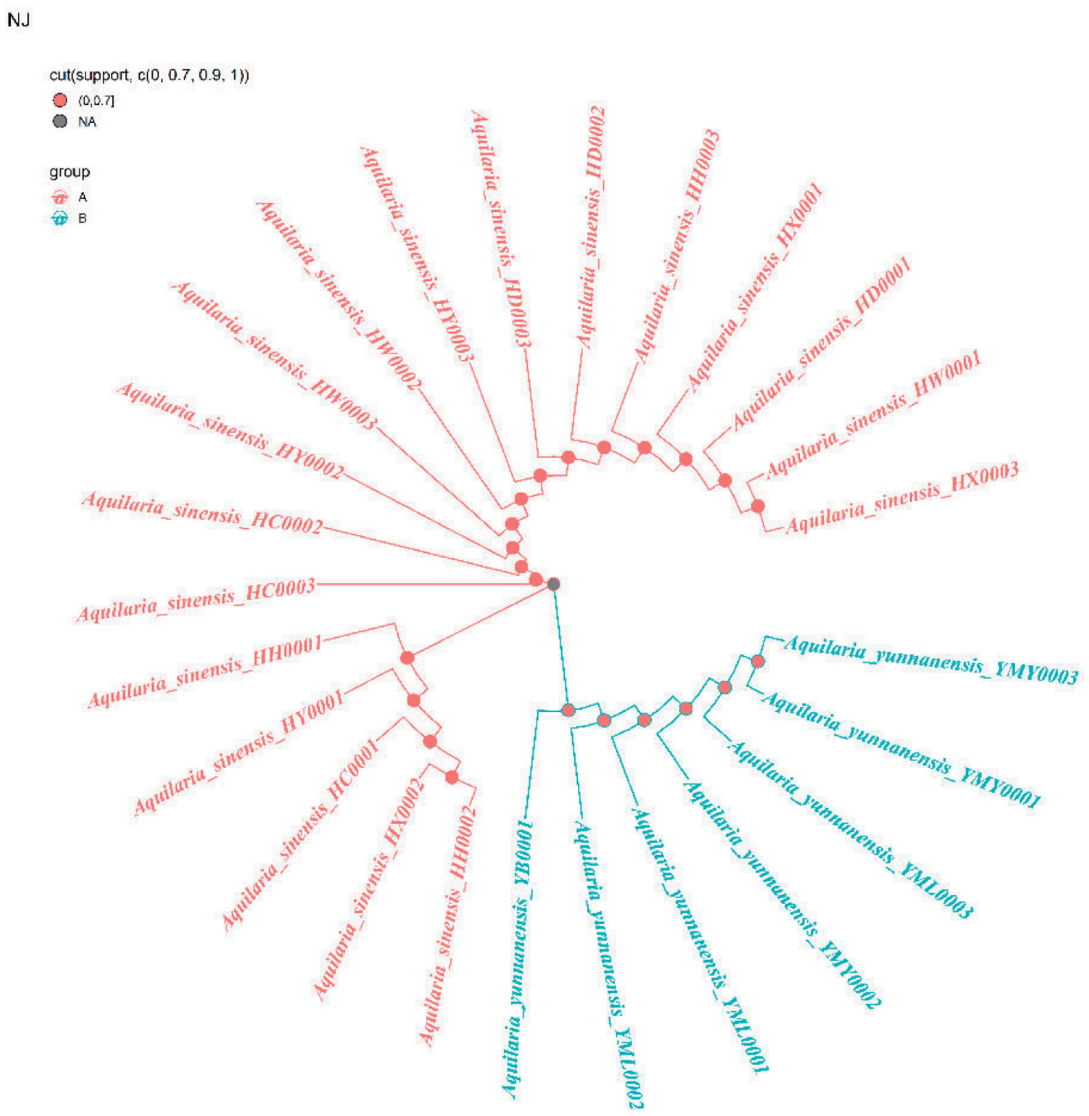
Construction of NJ trees of *A. sinensis* and *A. yunnanensis* using ITS2+*matK*.

**Figure 4.**
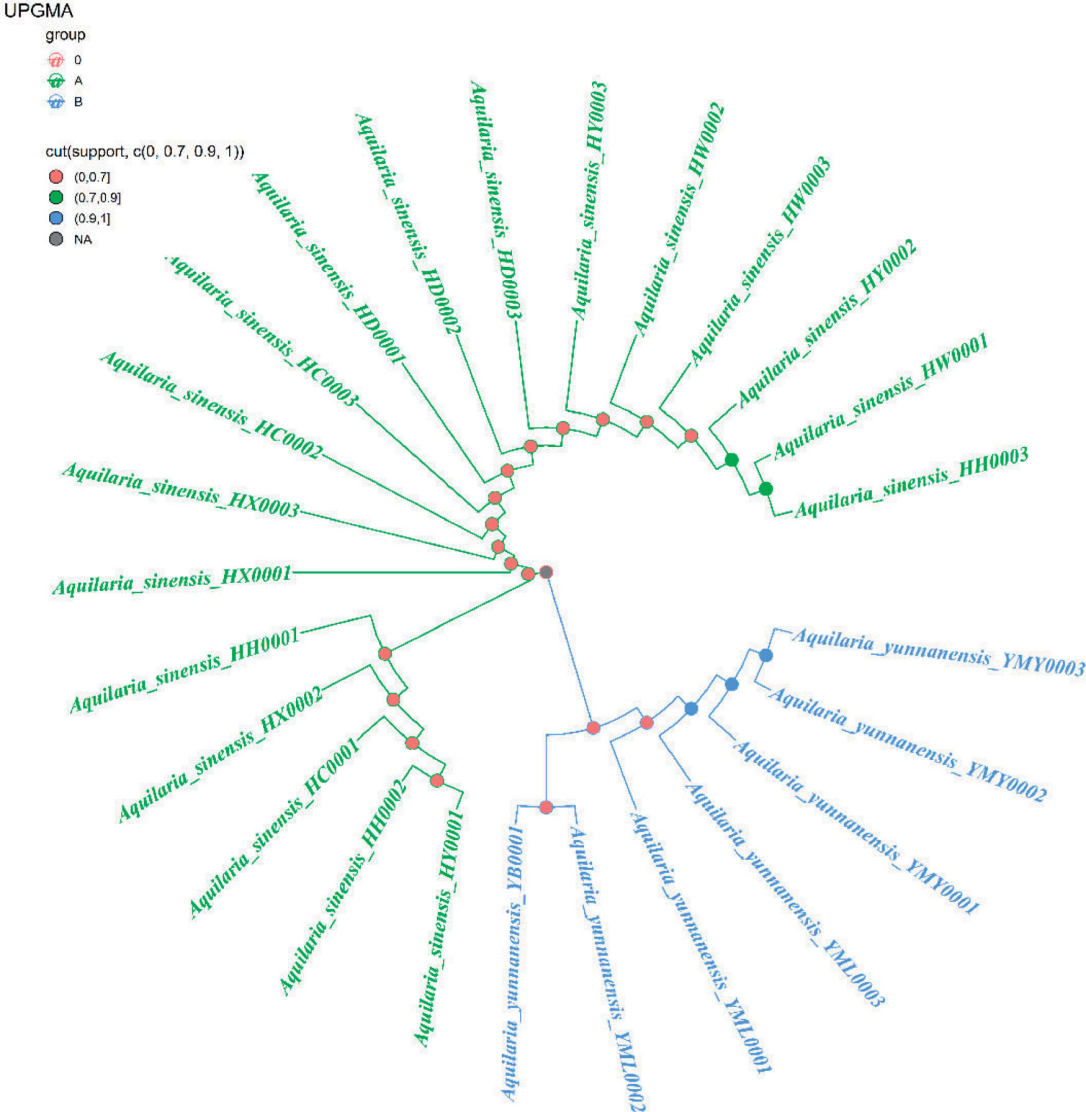
Construction of UPGMA trees of *A. sinensis* and *A. yunnanensis* using ITS2+*matK*.

**Figure 5.**
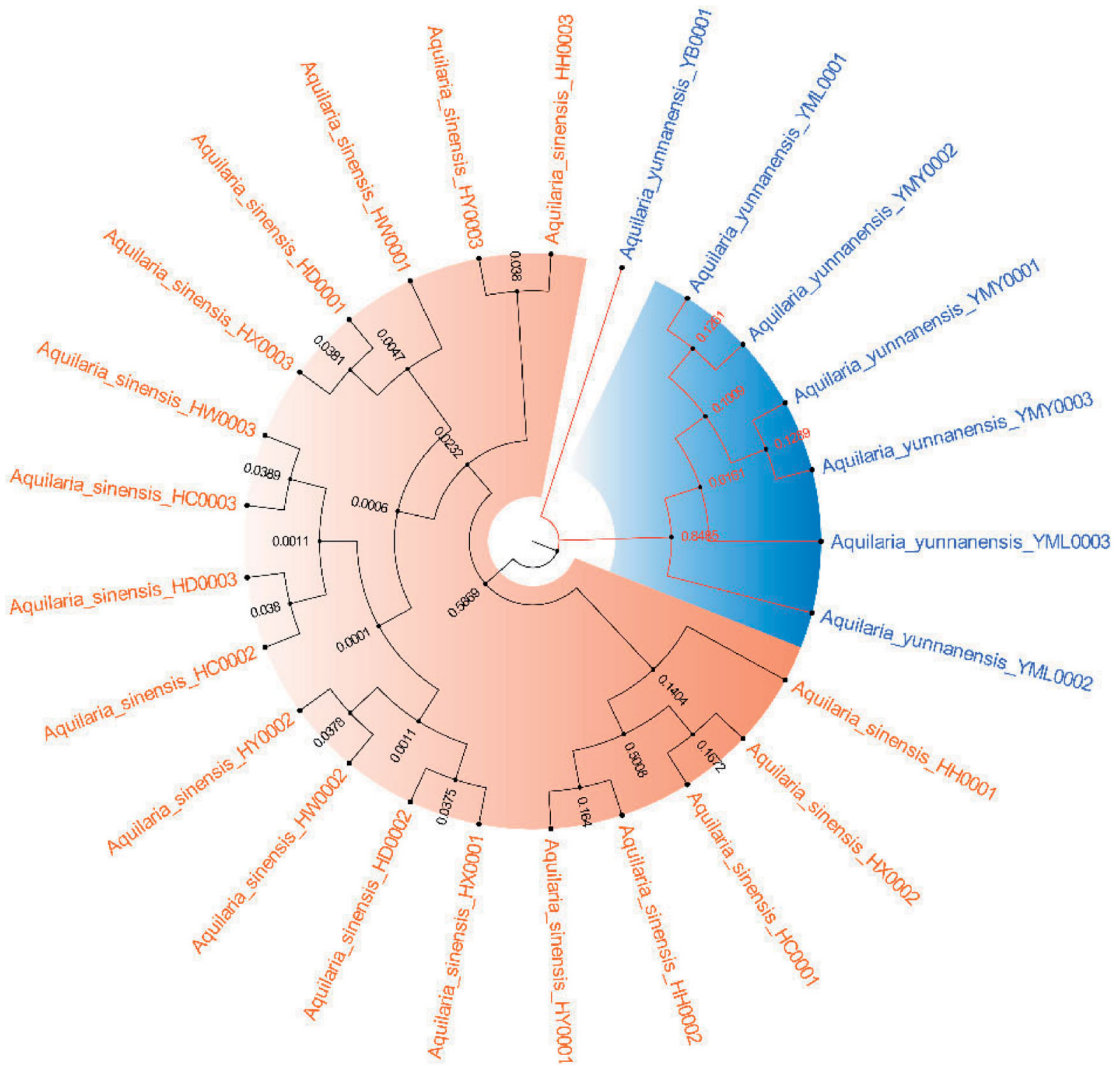
Construction of Bayes trees of *A. sinensis* and *A. yunnanensis* using ITS2+*matK*.

**Figure 6.**
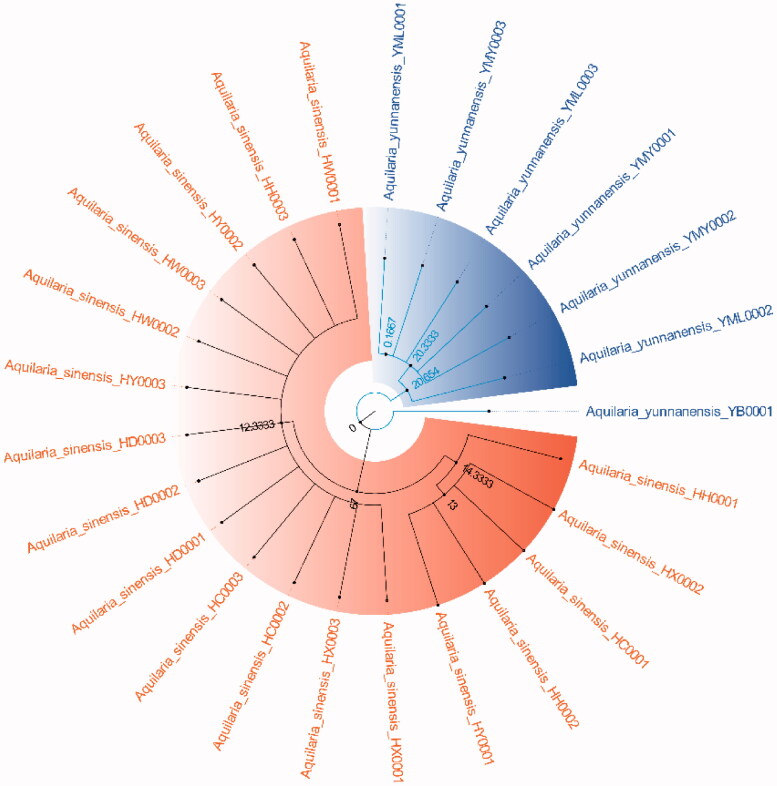
Construction of ML trees of *A. sinensis* and *A. yunnanensis* using ITS2+*matK*.

Because ITS2+*matK*, ITS2+*matK*+*trnl-trnf2*, ITS2+*matK*+*trnH-psbA* and ITS2+*matK*+*trnl-trnf2*+*trnH-psbA* have the highest success rates of species identification (96%) and the 2 fragment combinations are more convenient in the process of constructing phylogenetic tree, with the sequencing cost was low, so this study chose ITS2+*matK* combination to construct NJ, UPGMA, BI and ML phylogenetic trees respectively (Figures 3–6). The results showed that the phylogenetic trees constructed by the four methods can clearly divide *A. sinensis* from Hainan and *A. yunnanensis* from Yunnan, with significant geographic isolation between the two species.

## Discussion

### DNA barcoding evaluation of *Aquilaria* species in China

This study used five primers (i.e. ITS2, *matK*, *trnL-trnF1*, *trnL-trnF2* and *trnH-psbA*) to analyze the DNA barcodes of *A. sinensis* and *A. yunnanensis* (Table 2). In 2009, *rbcL* and *matK* were proposed officially as universal barcodes for terrestrial plants by CBOL research team. Then, ITS and *trnH-psbA*, which demonstrate faster evolutionary rates, were suggested as candidate barcodes by all participants in the 3^rd^ International Academic Conference of DNA Barcode (Group et al. 2009). That is, ITS, *matK, rbcL* and *trnH-psbA* are considered as common DNA barcodes. However, the coding sequence of *rbcL* is highly conserved, leading its variation mainly exists at the level of genus or above and is usually small at the species level (Newmaster et al. 2006; Kress and Erickson 2007; Lahaye et al. 2008). By filtering DNA barcodes from medical biology and analyzing 6600 ITS2 sequences of 4800 algae, fungus and higher plants under 753 genera, Chen Shilin’s team found that the resolution success rate of ITS2 at the species level is 92.7%. They proposed that ITS2 could be used as a new DNA barcode for fungi and green plants (Chen et al. 2010). Furthermore, ITS2 has a shorter sequence length than ITS, and its high rates of PCR amplification or sequencing (Wang et al. 2016). Meanwhile, *trnL-trnF* was also found that plays an important role in molecular identification for *Aquilaria* species (Eurlings and Gravendeel 2005). Therefore, ITS2, *matK*, *trnH-psbA*, *trnL-trnF1* and *trnL-trnF2* were selected for the identification of *Aquilaria* species in this study.

By comparing the sequence characteristics of different primers and analyzing their genetic distances and species identification rates, we found that the ITS2+*matK* barcode combination has the highest species identification success rate (96%), which could be apply to identification and phylogenetic tree construction of *A. sinensis* and *A. yunnanensis* (Figures 3–6). DNA barcoding has been widely for identifying *Aquilaria* species in nowadays. For example, Jiao et al. found that the significant clustering effect of phylogenetic tree construction by *trnL-trnF* and ITS1 for *A. sinensis* (Jiao et al. 2014). When analyzing *Aquilaria* species, Lee et al. concluded that the phylogenetic tree constructed with combination ITS2+*trnL-trnF* was applicable for *Aquilaria* species (Lee et al. 2016). Li et al. found that the phylogenetic tree constructed by ITS+*matK* and ITS+*trnL-trnF* is beneficial to the identification of three *Aquilaria* species (Li et al. 2018). In this study, our results are differrent from those previous studies due to different fragments or combinations tend to have different identification effects for various *Aquilaria* species. Although the *trnL-trnF1* sequence showed significant intraspecific and interspecific genetic distances, the fragment and its combination displayed low species identification rates (Table S2). This may result from some repeated sequences in *trnL-trnF1* sequencing results, which is not useful for constructing the phylogenetic tree. Moreover, *trnL-trnF2* is not applicable for phylogenetic tree construction in this study due to the short sequence length, insignificant genetic distance, and low species identification rate. By analyzing the *Aquilaria* species in different countries, our the research team found that the *matK* fragment plays an important role in identifying *Aquilaria* species (Kang et al. 2019). And *matK* (81%) also showed the highest species identification rate in this study. However, the combination of two fragments is more convenient to operate and reduce the cost of sequencing, and the *trnH-psbA* variable sites is low. (Kress et al. 2005). For these reasons, ITS2+*matK* was finally selected from the four combinations (i.e. ITS2+*matK* (96.00%), ITS2+*matK*+*trnL-trnF2* (96.00%), ITS2+*matK*+*trnH-psbA* (96.00%), ITS2+*matK*+*trnL-trnF2*+*trnH-psbA* (96.00%)) and used for the clustering analysis of phylogenetic tree for the *Aquilaria* species in this study.

### Morphological difference and study progress of A. sinensis and A. yunnanensis

This study found that *A. sinensis* has either smooth surface or is grown with sparse hair and long appendages on its seeds through field investigation, while *A. yunnanensis* has dense yellow pubescence and short appendages on its seeds, which are consistent with the main identification features as recorded in Flora of China. For example, the texture of the capsule of *A. sinensis* is slightly thin, the skin does not shrink when it is dry, the seeds are white silky or glabrous, the apex has a long beak, the base appendage is longer, about 1.5 cm, longer than the seed. However, *A. yunnanensis* has thick capsules, shriveled dry pericarps, yellow pubescence on the seeds, short floral organs on top, and short base appendages that are almost equivalent with that of the seeds, with a length of about 0.8–1 cm (FOC Eco 1999). In addition, *A. sinensis* is mainly distributed in Guangdong (including HK and Macao), Hainan and Guangxi, while *A. yunnanensis* is mainly distributed in Xishuangbanna, Yunnan (Huang 1985).

The phylogenetic tree that constructed by ITS2+*matK* combination has clearly branched out *A. sinensis* and *A. yunnanensis* (Figures 3–6), showing their distant genetic relationship, which is speculated to have resulted from geographical isolation. At present, the research of *A. sinensis* has involved molecular, genetic, microscopic, chemical and aromatic aspects (Liu Y et al. 2013; Liu P et al. 2019; Sun et al. 2020; Wang J et al. 2019; Wang Z et al. 2020). *A. sinensis* is a unique source of domestic agarwood according to Chinese Pharmacopeia (Committee SP 2020). However, there are relatively scarce studies that focus on *A. yunnanensis*. Although they are greatly different in terms of the morphological features of seeds or fruits, the anatomical structures of *A. yunnanensis* are basically consistent with *A. sinensis*, with endophloem scattered in its xylem (Su et al. 2016). This enables *A. yunnanensis* to generate agarwood for making agarwood for medicine or spice. However, whether there are differences in the regulatory genes or chemical components of the two *Aquilaria* species still needs further study in the process of forming agarwood. And there is a new record of *A. yunnanensis* in Vietnam (Van Sam et al. 2019).

Most *Aquilaria* species are distributed mainly in tropical or subtropical regions of Southeast Asia, leading in great challenges for taxonomical study. However, DNA barcode technology can ensure the reliability of the results when the sampling is accurate and covers a wide range of distribution (Ren and Chen 2010). Therefore, traditional taxonomy is essential for accurately collecting and identifying the *Aquilaria* species. Moreover, the investigation and collection of *Aquilaria* species is the key to identify the original species of agarwood, and this work can promote the stability of agarwood market.

## Data Availability

The data that support the findings of this study are openly available in NCBI GenBank database at (https://www.ncbi.nlm.nih.gov), and the reference numbers [MW118060-MW118109, MW124309-MW124383] are shown in Table 1.
